# Abdominoplasty Skin-Based Dressing for Deep Wound Treatment—Evaluation of Different Methods of Preparation on Therapeutic Potential

**DOI:** 10.3390/pharmaceutics13122118

**Published:** 2021-12-08

**Authors:** Dawid Groth, Izabela Poplawska, Marlena Tynecka, Kamil Grubczak, Jordan Holl, Aleksandra Starosz, Adrian Janucik, Klaudia Borkowska, Dorota Juchniewicz, Hady Razak Hady, Slawomir Czaban, Joanna Reszec, Artur Kaminski, Tomasz Czech, Cezary Kowalewski, Piotr Fiedor, Zbigniew Zimek, Hanna Lewandowska, Tomasz Oldak, Marcin Moniuszko, Andrzej Eljaszewicz

**Affiliations:** 1 Department of Regenerative Medicine and Immune Regulation, Medical University of Bialystok, 15-269 Bialystok, Poland; dawid.groth@umb.edu.pl (D.G.); marlena.tynecka@umb.edu.pl (M.T.); kamil.grubczak@umb.edu.pl (K.G.); jordan.holl@umb.edu.pl (J.H.); aleksandra.starosz@umb.edu.pl (A.S.); adrian.janucik@umb.edu.pl (A.J.); klaudia.borkowska@umb.edu.pl (K.B.); dorota.juchniewicz@umb.edu.pl (D.J.); 2 Eastern Poland Burn and Reconstructive Surgery Center, Department of Plastic Surgery, District Hospital in Leczna, 21-010 Leczna, Poland; 3 Department of Medical Pathomorphology, Medical University of Bialystok, 15-269 Bialystok, Poland; izabela.oplawska@umb.edu.pl (I.P.); joanna.reszec@umb.edu.pl (J.R.); 4 Department of General and Endocrinological Surgery, Medical University of Bialystok, 15-276 Bialystok, Poland; hadyrazakh@wp.pl; 5 Department of Anaesthesiology and Intensive Therapy, Faculty of Health Sciences, Medical University of Bialystok, 15-276 Bialystok, Poland; slawomir.czaban@umb.edu.pl; 6 Department of Transplantology and Central Tissue Bank, Medical University of Warsaw, 10-002 Warsaw, Poland; artur.kaminski@wum.edu.pl; 7 National Centre for Tissue and Cell Banking, 02-004 Warsaw, Poland; czech_t@prokonto.pl; 8 Department of Dermatology and Immunodermatology, Medical University of Warsaw, 10-002 Warsaw, Poland; ckowalewski@wum.edu.pl; 9 Department of General and Transplantation Surgery, Medical University of Warsaw, Infant Jesus Clinical Hospital, 02-005 Warsaw, Poland; piotrfiedor@wp.pl; 10 Institute of Nuclear Chemistry and Technology, 03-195 Warsaw, Poland; z.zimek@ichtj.waw.pl (Z.Z.); h.lewandowska@ichtj.waw.pl (H.L.); 11 Polish Stem Cell Bank (PBKM), 00-867 Warsaw, Poland; Tomasz.Oldak@pbkm.pl; 12 Department of Allergology and Internal Medicine, Medical University of Bialystok, 15-276 Bialystok, Poland

**Keywords:** skin substitutes, human acellular dermal matrices, hADMs, wound healing, dermal grafts

## Abstract

The management of hard-to-heal wounds is a significant clinical challenge. Acellular dermal matrices (ADMs) have been successfully introduced to enhance the healing process. Here, we aimed to develop protocol for the preparation of novel ADMs from abdominoplasty skin. We used three different decellularization protocols for skin processing, namely, 1M NaCl and sodium dodecyl sulfate (SDS, in ADM1); 2M NaCl and sodium dodecyl sulfate (SDS, in ADM1); and a combination of recombinant trypsin and Triton X-100 (in hADM 3). We assessed the effectiveness of decellularization and ADM’s structure by using histochemical and immunochemical staining. In addition, we evaluated the therapeutic potential of novel ADMs in a murine model of wound healing. Furthermore, targeted transcriptomic profiling of genes associated with wound healing was performed. First, we found that all three proposed methods of decellularization effectively removed cellular components from abdominoplasty skin. We showed, however, significant differences in the presence of class I human leukocyte antigen (HLA class I ABC), Talin 1/2, and chondroitin sulfate proteoglycan (NG2). In addition, we found that protocols, when utilized differentially, influenced the preservation of types I, III, IV, and VII collagens. Finally, we showed that abdominoplasty skin-derived ADMs might serve as an effective and safe option for deep wound treatment. More importantly, our novel dressing (ADM1) improves the kinetics of wound closure and scar maturation in the proliferative and remodeling phases of wound healing. In conclusion, we developed a protocol for abdominoplasty skin decellularization suitable for the preparation of biological dressings. We showed that different decellularization methods affect the purity, structure, and therapeutic properties of ADMs.

## 1. Introduction

Despite the development of new surgical techniques, dressings, and experimental therapies, the treatment of full-thickness wounds and non-healing ulcers remains challenging [1,2,3]. To date, the general procedure of treatment includes surgical wound preparation and its subsequent closure [4]. However, the preparation of wounds that involve all skin layers (when damage extends below the epidermis and dermis) requires a more complex approach. Consequently, in many cases, a full- or split-thickness skin autograft represents a beneficial therapeutic option [5]. Despite this, limitations in regard to both the quantity of available autologous skin as well as the subsequent complications associated with the invasive nature of skin autograft harvest constitute significant problems with this method.

Currently, acellular grafts—namely, allogenic acellular dermal matrices (ADMs)—are considered safe and abundant alternatives to autologous skin grafts for the therapeutic treatment of extensive wounds [6]. ADMs are a processed dermal biomaterial derived from skin fragments that have been chemically and/or enzymatically processed to remove all epidermal and dermal cellular components while preserving three-dimensional structures containing collagen, elastin, and bioactive proteins, among others [6]. Currently, commercially available ADMs, such as Integra, DermACELL, Apligraf, and Alloderm, have been tested mainly for their ability to treat burns, ulcers, and deep wounds, and they have also been used in reconstructive surgery with great success [7,8]. Despite the beneficial therapeutic effects induced by ADM application, the direct effects of different dermal matrixes on wound healing kinetics remain elusive. However, it is well established that both allo- and xenogeneic ADMs support the healing process by covering the wound, decreasing the frequency of wound infections, acting as a scaffold for various regenerative cell types during the wound healing proliferation phase and supporting the re-vascularization process [8,9,10]. Notably, xenogeneic ADMs (mostly derived from bovine or porcine bladder and intestine) possess limited application in patients due to allergic reactions to xenogeneic collagens as well as religious considerations [11]. Therefore, there exists a need to develop novel ADMs which may simultaneously increase the effectiveness of therapeutic support in the wound healing process while minimizing the chance of allergic reactions and patient concern.

Notably, the principal limitation of ADM generation is reliant upon accessibility to skin suitable for ADM preparation. Currently, the utilization of human cadaver-derived skin represents the principal ethically acceptable source of therapeutically applied ADMs [6]. Here, we proposed an alternative approach of using skin from abdominoplasty for the development of a novel human ADMs. Abdominoplasty (also known as a tummy tuck) is a popular cosmetic surgery procedure applied to remove the excess skin and adipose tissue around the abdomen. Given the ubiquity of abdominoplastic surgery in the general population, resected skin folds represent a readily available source of ADMs. Therefore, here we aimed to develop a protocol for the preparation of novel ADMs from abdominoplasty skin. Furthermore, given the differential healing effect that can be induced by the ADM preparation method, we compared different decellularization protocols and analyzed the effectiveness of each process, effects on collagen structure, and therapeutic potential in vivo [8,12].

## 2. Materials and Methods

### 2.1. Skin Collection and Biobanking

Skin fragments were collected from post-bariatric surgery patients classified to an abdominoplasty. Before surgery, patients were examined, and those who met the criteria for a full abdominoplasty with umbilical transposition (type IV by Matarasso) were included in the study. All procedures were undertaken with systemic anesthesia. The surgeries were performed by the same operator and two assistants, as previously described [13]. Briefly, an incision was made from hip to hip, above the pubic area. Then, the abdominal flap was dissected in a preaponeurotic plane to the level of the subcostal margin. The navel was dissected. The dissection was performed with a diathermocoagulation device. Two Redon drains were used in all patients, one on each side of the lower abdomen. The excised skin fold was removed, and skin grafts were collected immediately using an electric dermatome (Aesculap Acculan 3Ti, Braun, Frankfurt, Germany). Finally, the samples were sealed in double foil bags and biobanked in −80 °C for further processing.

All skin fragments were collected after receiving approval from the Ethics Committee at the Medical University of Bialystok. Study participants gave written informed consent, and the study was conducted following the provisions of the Helsinki Declaration.

### 2.2. Decellularization Procedures

To find the most effective ADM preparation method from abdominoplasty skin, three different decellularization methods (Table 1) were used. They are here referred to as Human Acellular Dermal Matrix-1 (ADM1–1M NaCl + SDS), -2 (ADM2–2M NaCl + SDS), and -3 (ADM3–TrypLE Select + Triton X-100).

Briefly, skin fragments were thawed in prewarmed saline (Biomed Lublin, Lublin, Poland) and washed two times. Next, skin was immersed in 1M NaCl (Sigma, ADM1, St. Louis, MO, USA), 2M NaCl (Sigma, ADM2), or TrypLE Select (Thermofisher, ADM3, Waltham, MA, USA) and incubated for 24 h at 37 °C with gentle agitation. Next, the epidermis was removed by tweezers (ADM1 and ADM2). After trypsinization (ADM3), the epidermis was detached and cleaved without mechanical removal. Next, the grafts were washed in buffered saline (PBS without magnesium and calcium ions, Corning, Corning, NY, USA) supplemented with antibiotics and subjected to the second step procedure. ADM1 and ADM2 were incubated for 24 h in buffered 0.5% sodium dodecyl sulfate (SDS, Sigma) at 37 °C, while ADM3 was incubated in 3% Triton X-100 (Sigma) for 24 h at 37 °C. Next, matrices were washed for five days in sterile ddH2O with daily water changes. Finally, all ADM were freeze-dried for 12–24 h, sealed in double foil bags and subsequently irradiated in electron accelerator with a dose of 35 kG at the Institute of Nuclear Chemistry and Technology Warsaw. The sterilized ADMs were stored at −80 °C for later usage.

### 2.3. Histochemical and Immunohistochemical Staining

The presence of different cellular components and composition of different collagen structures was assessed by histochemical staining Six-millimeter biopsy-punched lyophilized and irradiated scaffold fragments were rehydrated in PBS (Corning) for 24 h. The scaffolds were then fixed in 4% paraformaldehyde and paraffin-embedded using a tissue processor (Xpress Sakura). Then, 4 μm microtome slices were placed on glass slides (ThermoScientific, Waltham, MA, USA) and stained with hematoxylin-eosin to assess the decellularization process’s effectiveness. For whole collagen structure visualization, Masson’s Trichrome staining was used. A detailed scaffold structure analysis was performed based on immunohistochemical staining, as described previously. For detailed information on the antibodies used, please see Supplementary Table S1.

### 2.4. Quantification of Immunohistochemical Staining

The presence of particular components of the extracellular matrix was quantified using ImageJ software (NIH). Three different values were assessed in all microscopic slides. To measure the surface area of tissue and collagen surface color, a threshold tool was used. The slider in the brightness panel was adequately adjusted to cover all tissue areas. The default thresholding method and the HSB model for color space were selected to perform the analysis. Additionally, a slider for the hue panel was acquired to incorporate the range of orange–red colors. For tissue surface measurements, the values in the hue and saturation panels were selected, as dedicated initially by the software. Finally, the total microscopic slide area was measured. All values were saved in the results window and exported to an Excel file. The presented data are the measurements in the scaffold area.

### 2.5. Peripheral Blood Mononuclear Cells Isolation

Allogenic peripheral blood mononuclear cells (PBMCs) from freshly obtained EDTA-anticoagulated blood were isolated by density-gradient centrifugation with Histopaque-1077 (Sigma-Aldrich). Cells were subsequently washed and quantified using a Bürker chamber slide. The material was collected after receiving approval from the Bioethical Committee of the Medical University of Bialystok. Each participant was familiarized with the objectives of the study and expressed written consent for material collection.

### 2.6. Proliferation Assay

To assess ADM immunogenicity, a CFSE-stained T cell proliferation assay was used. Briefly, two million freshly isolated PBMCs were labeled with CFSE and incubated in 2 mL of RPMI1640 medium with stable glutamine (PAN Biotec, Aidenbach, Germany) supplemented with 10% FBS (PAN Biotec) and gentamycin (Thermofiher), in the presence of full human unprocessed skin (positive control), ADM1, ADM2, ADM3, phytohemagglutinin (PHA, proliferation control, R&D Biosystem) or the vehicle alone (negative control). After a seven-day incubation at 37 °C and 5% CO_2_, the cells were washed and subsequently stained with anti-CD4 PerCP-conjugated monoclonal antibodies (clone MEM-241, Becton Dickinson Bioscience, Franklin Lakes, NJ, USA) for 30 min at room temperature in the dark. Finally, samples were analyzed on FACSCalibur flow cytometer (Becton Dickinson Bioscience). Data analysis was performed using FlowJo software (TreeStar, Ashland, OR, USA).

### 2.7. Murine Model of Wound Healing

Twelve healthy 8-week-old female Balb/ccmdb SPF mice were used in this study. Animals were obtained from the Centre for Experimental Medicine of the Medical University of Bialystok. The mice were maintained in a temperature-controlled environment (22 +/−1 °C), controlled humidity (45–55%), with a 12 h light–dark cycle beginning at 7 a.m., and were housed in polycarbonate cages, with access to water and food (Labofeed H Standard, Morawski, Poland) available ad libitum. The murine model of wound healing was applied as previously described [14]. Briefly, one day before surgery, the mouse dorsum was shaved with an electric razor. Animals were anesthetized with 1% isoflurane. While anesthetized, two 4 mm full-thickness round cutaneous wounds were created with a manual biopsy punch. Silicone rings were anchored around wounds using 6-0 nylon suture (Huaiyin Medical Instruments Co., Ltd., Huaian, China) to prevent wound irritation and the contribution of murine wound contraction to wound healing kinetics. A 4 mm PBS-rehydrated ADM was placed directly on the wound within the silicone ring. The animals were divided into three groups ADM1, ADM2, and ADM3, depending on the processed ADM applied to the 1st dorsal wound. The 2nd dorsal wound was left without any intervention as a control (healing by granulation). The rings were covered with transparent Tegaderm film (3M Health Care, Saint Paul, MN, USA), and the mice were placed in separate cages. Postoperative analgesia was provided with subcutaneous injection of ketoprofen (Vetaprofen, KELA N.V., Antwerpen, Belgium)—5 mg/kg s.c. once per day. Wound measurements (performed based on images of the wound) were performed daily after brief anesthesia followed by transparent Tegaderm film changes. Quantification of wound closure was performed using ImageJ software (NIH). On day 14, mice were sacrificed by isoflurane overdose and subsequent cervical dislocation, followed by scar tissue acquisition using a 4 mm biopsy punch. Tissue samples were immersed in RNAlater solution (Thermofisher) and biobanked at −80 °C for further analysis. All procedures were performed under laminar flow with sterile instruments according to the excisional wound splitting model [15]. The study was approved by the Local Ethical Committee in Olsztyn, Poland.

### 2.8. RNA Isolation and Quantitative PCR Assay

The wound scars were homogenized with TissueRuptor (Qiagen, Hilden, Germany) in lysing buffer. Wound mRNA was purified through the RNeasy Mini kit (QIAGEN), according to the manufacturer’s instructions. The RNA was quantified on NanoDrop (Thermofisher), and the integrity was analyzed on a bioanalyzer (Agilent, Santa Clara, CA, USA). Next, 1 μg of mRNA from each sample was reverse transcribed with a high-capacity cDNA reverse-transcription kit (Thermofisher) according to the manufacturer’s instructions. The expression level of 90 genes associated with wound healing was assessed by using PrimePCR Wound healing assay according to the manufacturer’s instructions (BioRad, Hercules, CA, USA). The relative expression of 88 genes associated with the healing process was calculated and normalized to GAPDH expression. All presented data were normalized to the control by using the 2^-ddCt method and presented as log2FC.

### 2.9. Statistics

Data analysis was performed with GraphPad Prism v7 software (GraphPad Software). Differences between analyzed groups were evaluated by using the Student’s *t*-test. The differences were considered statistically significant at *p* < 0.05. The results are presented as mean ± standard deviation.

## 3. Results

### 3.1. Abdominoplasty Skin Is Suitable for Human Acellular Dermal Matrix Preparation

Here, we used three different methods (Table 1) of decellularization for abdominoplasty skin processing. The preparation of ADM1 and ADM2 started with the application of 1M- or 2M NaCl, respectively, to detach the epidermis. Following this, both ADM1 and ADM2 were exposed to a 0.1% SDS solution to induce decellularization. In contrast, the ADM3 protocol utilized a combination of enzymatic cleavage and chemical washing—namely, by subsequent incubation periods with recombinant trypsin and 3% Triton X-100. Next, all ADMs underwent a 5-day washing period with daily H_2_O change and freeze drying. Finally, all matrices were subjected to radiation sterilization according to the optimized protocol.

**Figure 1:**
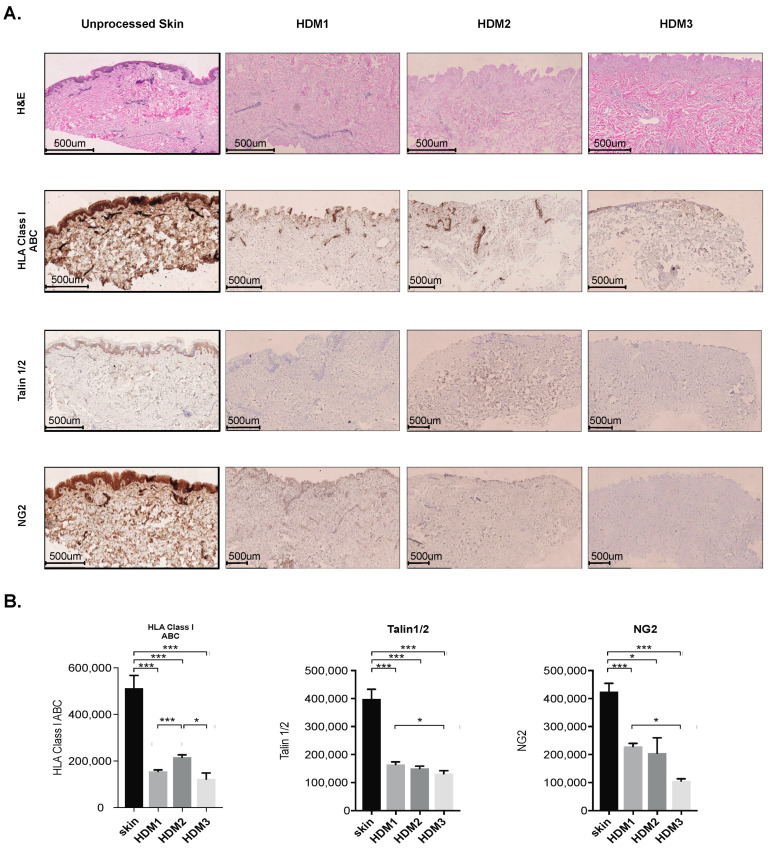
Effective decellularization of abdominoplasty skin. (**A**) Representative histochemical (H&E) and immunohistochemical staining (HLA Class I ABC, Talin 1/2, and NG2 proteoglycan) of ADMs derived from abdominoplasty skin decellularized with ADM1, ADM2, or ADM3 protocol. Representative pictures of five independent analyses. (**B**) Summary of quantification analyses of immunohistochemical staining for HLA Class I ABC, Talin 1/2, and NG2 proteoglycan in abdominoplasty skin and ADMs derived from abdominoplasty skin decellularized with ADM1, ADM2, or ADM3 protocol (n = 5). Size bar—500 μm; The Student’s *t*-test was used. * *p* < 0.05; *** *p* < 0.001.

First, by using H&E staining (Figure 1A), we found that all three methods used for abdominoplasty skin decellularization allowed cells to be effectively removed from the collagen structure. Next, the effects of different decellularization protocols were analyzed to assess their ability to eliminate cellular components which may orchestrate immunogenicity and influence therapeutic potential (Figure 1A). Interestingly we found that ADM1 and ADM2 protocols were less efficient in removing HLA Class I group proteins (Figure 1B), which is especially noticeable in the apical regions and former vein locations. In contrast, the ADM3 protocol resulted in a significant decrease in the presence of these molecules. Somewhat surprisingly, we observed that the higher concentration of NaCl, used in the ADM2 protocol, significantly preserved Talin 1/2 when compared to both ADM1 and ADM3 (Figure 1B). In contrast, NG2 was highly preserved in ADM1, while the remaining two matrices presented significantly lower levels of this proteoglycan. Importantly, we found that ADM1 is less immunogenic when compared to the remaining two matrices (Supplementary Figure S1).

### 3.2. Decellularization of Abdominoplasty Skin Preserves Collagen III and IV Architecture

**Figure 2:**
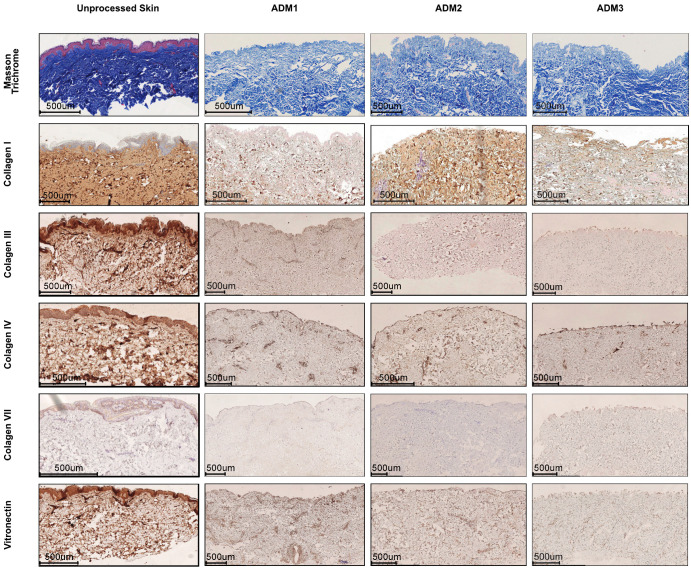
Different decellularization protocols preserve matrix structure but change the presence of collagen fibers. Representative histochemical (Masson Trichrome) and immunohistochemical (Collagen I, II, III, IV, VII, and Vitronectin) staining of ADMs derived from abdominoplasty skin decellularized with ADM1, ADM2, or ADM3 protocol. Representative pictures of five independent analyses. Size bar—500 μm.

Having found that all examined decellularization procedures were suitable for ADM preparation, we wished to analyze whether applied protocols may affect the extracellular matrix structure. First, based on visualization of Masson’s Trichrome-stained fragments, we found that all decellularization protocols preserved characteristic dermal collagen structure. Furthermore, detailed analysis revealed that the ADM2 retained a higher density of collagen I fibers, while ADM1 retained a higher collagen III density (Figure 2 and Supplementary Figure S2). Similarly, to collagen III, collagen IV was highly preserved after decellularization with the ADM1 protocol. Surprisingly, however, we observed that all applied decellularizations significantly reduced collagen VII fibers in the apical region of ADMs (Figure 2). Finally, we found that all used protocols preserved the abundance of vitronectin (Figure 2) in the ADM structure, with the highest levels observed in ADM1.

### 3.3. Abdominoplasty Skin-Derived ADM Accelerates Wound Closure

**Figure 3:**
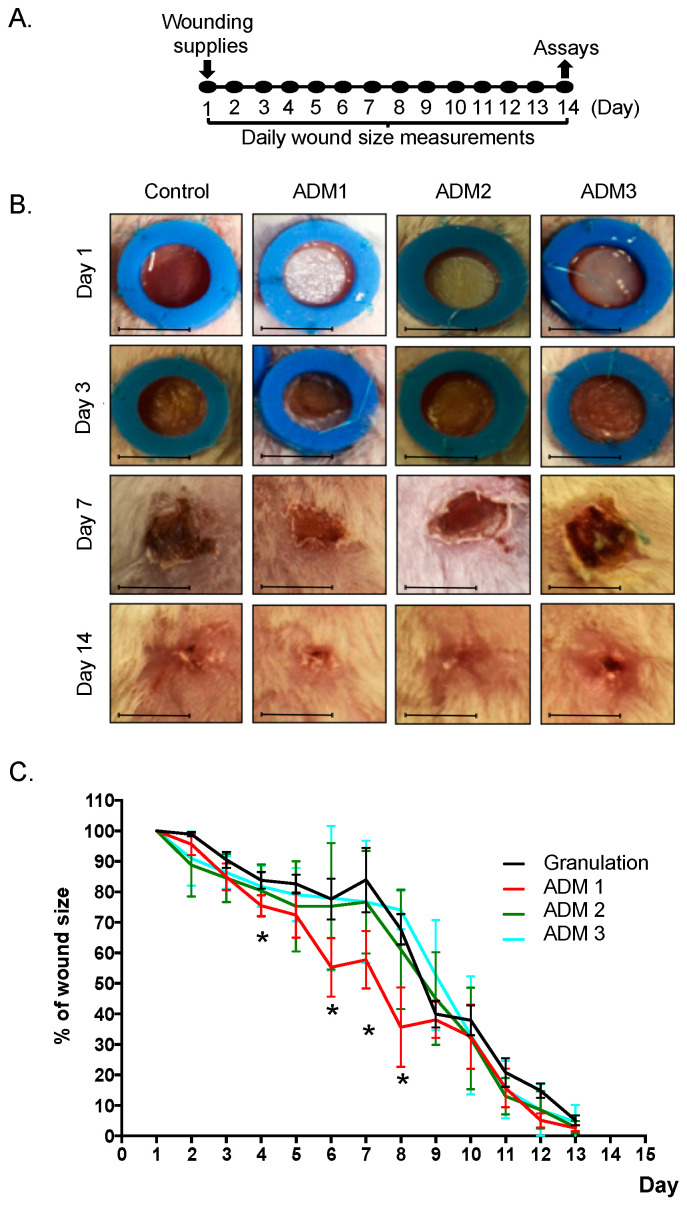
Acellular Dermal Matrix 1 (ADM1) enhances the proliferative phase of wound healing. (**A**) Murine model of wound healing. A rehydrated ADM was placed directly on the wound within the silicone ring. The animals were divided into three groups, depending on the processed ADM applied, namely, ADM1, ADM2, and ADM3. Untreated wound served as a control. Wound measurements were performed daily. (**B**) Representative pictures of wounds at Day 1 (inflammatory phase), Day 2 (onset of proliferation phase), Day 7 (termination of proliferation phase), and Day 14 (maturation phase). (**C**) Summary of quantification of wound closure. n = 5; size bar—4 mm (initial wound size); *—Granulation vs. ADM1 *p* < 0.05; The Student’s *t*-test was used.

Having found differences in the composition of extracellular matrix components, we aimed to assess the therapeutic potential of each ADM. For this purpose, a murine wound healing model was used (Figure 3A). Wounds were covered immediately with ADMs following wound induction on day one, with subsequent daily wound size measurements for 13 consecutive days (Figure 3B). We found no differences in wound healing kinetics during the initial inflammatory phase of wound healing (day 1–3) for all analyzed conditions (Figure 3C). Interestingly, however, we found that wounds treated with ADM1 significantly resulted in increased wound closure kinetics during the proliferation phase (from day 4 to day 8), while remaining matrices showed normal (comparable to healing by granulation) closure kinetics. The most striking differences were observed at day 7 (before the release of silicone rings), with 42.2% overall closure for ADM1-covered wounds compared against 23.3% closure for ADM2- and ADM3-covered wounds, and 16.1% closure in control wounds. Interestingly, on day 9, the size of all analyzed wounds was similar, and no other differences were observed for the remainder of the 13-day study period (remodeling phase).

### 3.4. ADM1-Treated Wounds Show Slightly Modified Transcriptomic Profiles When Compared to ADM2- and ADM3-Treated Counterparts

**Figure 4:**
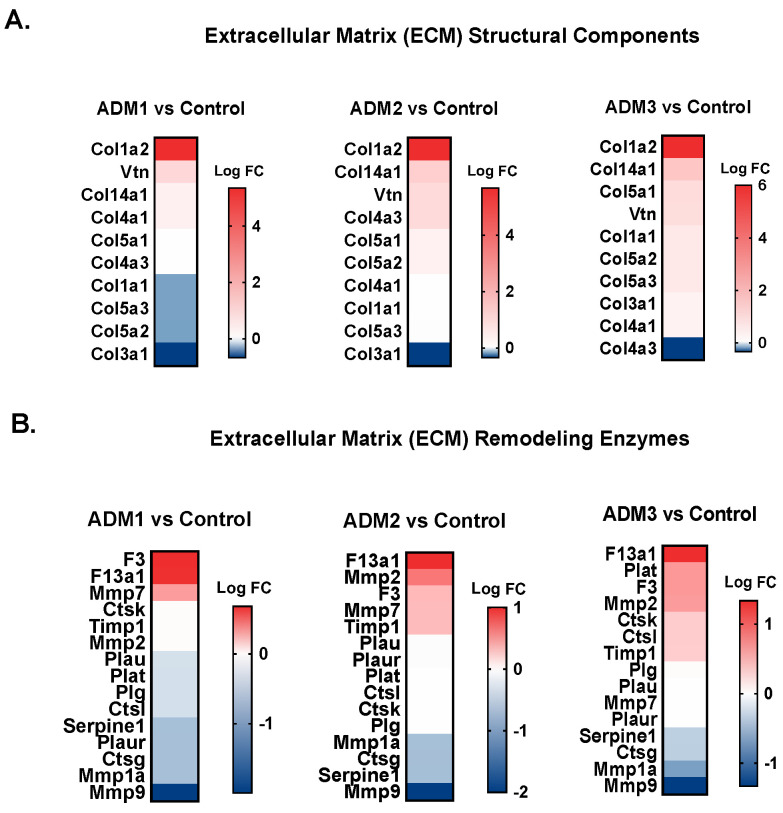
Targeted transcriptomic profiles of extracellular matrix components and remodeling enzymes in wounds treated with different acellular dermal matrices (ADMs). Summary of gene expression analyses of (**A**) extracellular matrix structural components and (**B**) extracellular matrix remodeling enzymes in wounds treated with ADM1, ADM2, and ADM3. The data are presented as log_2_FC (n = 3). For each presented heat map, a separate color scale legend with values can be found on the right side.

**Figure 5:**
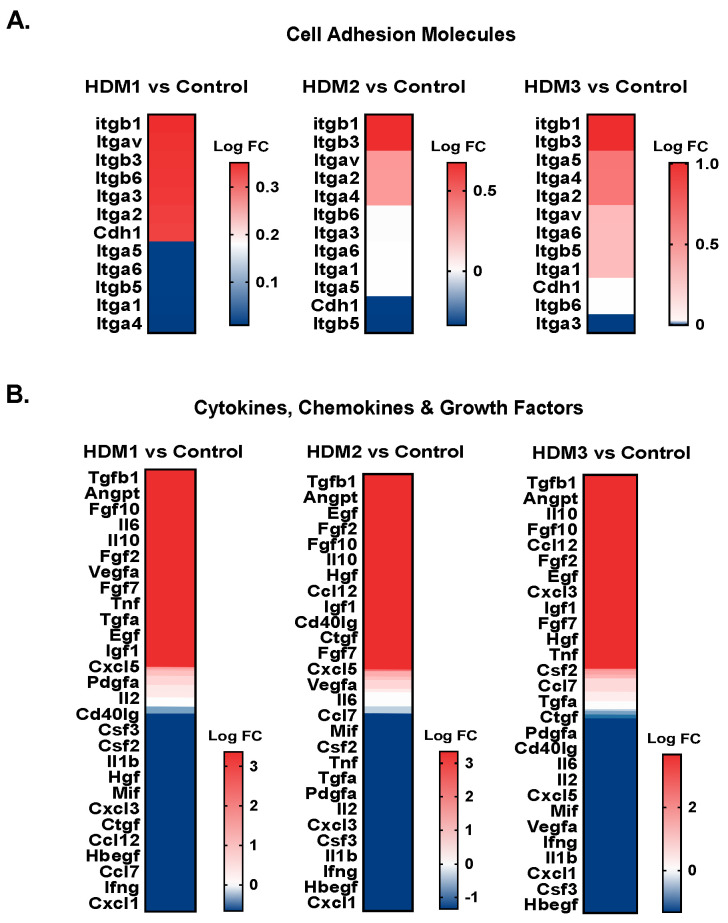
Targeted transcriptomic profiles of cell adhesion molecules and different soluble mediators in wound treated with different acellular dermal matrices (ADMs). Summary of gene expression analyses of (**A**) cell adhesion molecules, and (**B**) cytokines, chemokines, and growth factors in wounds treated with ADM1, ADM2, and ADM3. The data are presented as log_2_FC (n = 3). For each presented heat map, a separate color scale legend with values can be found on the right side.

Finally, to better understand the beneficial therapeutic potential associated with ADM1 in regard to its enhanced wound closure ability, we analyzed the influence of novel ADMs on the expression levels of genes associated with the healing process, namely extracellular matrix structural components (Figure 4A), extracellular matrix remodeling enzymes (Figure 4B), cell adhesion molecules (Figure 5A), and inflammatory mediators (cytokines, chemokines and growth factors, Figure 5B).

First, we found a similar pattern of upregulated genes in all ADM-treated wounds. However, we observed a trend to decrease in Col1a1, Col5a3, and Col5a2 gene expression in ADM1-treated wounds, while those treated with ADM3 possessed slightly upregulated expression (Figure 4A). Next, we found no striking differences in the expression signatures of extracellular matrix remodeling enzymes (Figure 4B). Despite this, differences in the expression signatures of cell adhesion molecules were observed (Figure 5A). Notably, ADM1-treated wounds had a slightly downregulated expression of Ifga1, while the same gene was upregulated in ADM3-treated wounds. Similarly, Itga4 expression was downregulated in ADM1-treated wounds, in spite of the fact that its expression was upregulated in ADM2 and ADM3. These differences in ECM and adhesion-related gene expression aside, we found intriguing differences in cytokine, chemokine, and growth factors gene expression levels (Figure 5B). Interestingly, expression of Vegfa was upregulated in ADM1- and ADM2-treated wounds and downregulated in ADM3 counterparts. Moreover, Csf3 was upregulated in ADM1-covered wounds, while it was downregulated in the ADM2 and ADM3 counterparts. Similarly, Ccl7 and Ccl12 genes were downregulated in ADM1 while being simultaneously upregulated in ADM2- and ADM3-covered wounds. We also observed upregulated expression of Il6 in ADM1-treated wounds with no changes in the remaining counterparts.

## 4. Discussion

Here, we demonstrated the capability of abdominoplasty skin to serve as a suitable material for ADM processing. We compared three different decellularization protocols by assessing their influence on extracellular matrix components, and immunogenicity. Moreover, by utilizing a murine model of deep wound healing, we evaluated the therapeutic potential of each of our novel ADMs. Importantly, we have shown that abdominoplasty skin processed with 1M NaCl and 0.5% SDS followed by washing steps, freeze drying, and radiation sterilization (ADM1) results in a novel biological dressing that improves wound closure kinetics.

The development of distinct decellularization methods has allowed organ-derived tissues to be used for tissue engineering and in successful clinical application [6,16,17,18,19,20]. To date, numerous differentially sourced mammalian- and plant-based decellularized tissues have been utilized for the development of biomimetic tissues which supplement the healing capability of the cardiovascular system, cartilage, bone, and skin [19,20]. Recently, our group and other groups, have reported the suitability of ADM for tissue reconstruction, burns, deep, and hard-to-heal wound treatment such as epidermolysis bullosa [12,21,22,23,24]. To date, several ADM-based products have been introduced to the market (such as Alloderm and GraftJacket); however, in contrast to our solution, all the available human sources of ADM are manufactured from cadaveric skin [6]. Abdominoplasty skin remains easily accessible, as the number of bariatric and abdominoplastic surgeries is growing proportionally with the observed global increase in severe obesity. In fact, the proposed skin source, similarly to cadaveric ones, may be processed according to the standards established by the American Association of Tissue Banks and routinely applied as a graft.

The principal role of a wound dressing is to cover the wound, which is essential for the initiation of the healing process, regardless of wound type. After wounding, the inflammatory reaction is induced and revealed by the infiltration of inflammatory cells to clean the wound of debris, damaged cells, and microbes [6]. However, the accumulation of inflammatory mediators may lead to prolonged inflammation and, consequentially, delayed wound repair characteristics for chronic wounds and ulcers [25]. Therefore, the ideal biological dressing should exert immunoregulatory properties to reduce local inflammation and, consequently, improve wound closure by promoting the proliferation phase. We found that different methods of abdominoplasty skin processing may affect this ability. In fact, we showed that our novel ADM1 improves the kinetics of wound closure in the proliferation phase (days 4–8) of wound healing. This therapeutic potential of ADM1 may be associated with (i) a limitation of the inflammatory phase, as increased wound closure was already observed at day 4, and (ii) the improvement in fibroblast and keratinocyte proliferation, as increased wound closure was observed in all analyzed time points of the proliferation phase. Based on our results and previous observations of intact collagen fiber contributions to wound healing, ADM’s therapeutic potential is heavily reliant on the composition of the matrix, which directly supports wound closure [26,27]. We found that ADM1 has a high presence of HLA class I ABC and NG2, as well as a relatively higher density of collagens III, IV, and vitronectin. Interestingly, the use of ionic and non-ionic detergents (such as SDS and Triton X-100, respectively) has been shown to be less destructive to extracellular matrix components when compared to enzymatic decellularization using enzymes with proteolytic activity (such as recombinant Trypsin, as in hADM3) [28,29,30,31]. These observations explain the higher levels of collagen III, IV, VII, and vitronectin observed in ADM3 compared to ADM1 and ADM2. On the other hand, SDS (an anionic detergent) is more effective in removing cellular components in the decellularization procedure [32,33]. However, in our study, we showed that the combination of recombinant trypsin and Triton X-100 was even more effective in removing NG2 and Talin 1/2. Furthermore, we showed that the use of different concentrations of NaCl in the first stage of the decellularization process might also affect the preservation of HLA class I ABC, which may be associated with a more problematic removal of the epidermis in the first stage of the process. HLA class I antigens are present in the vast majority of nucleated cells, including keratinocytes and fibroblasts. Interestingly, soluble (cell-released) MHC molecules have been shown to display immunomodulatory properties in two distinct ways, namely, directly (by binding physiological ligands and inhibit cytotoxic T-cell responses by TCR receptor blockage and/or induction of apoptosis [34,35]) or indirectly (phagocytosed by antigen-presenting cells, such as macrophages and dendritic cells, and presented in the context of MHC class II molecules to naïve T cells, potentially contributing to graft rejection [36]). However, the latter process may also induce tolerance to the presented immunogenic fragments depending on both antigens presenting cell- and HLA-derived peptide potential. HLA class I may also interact with NK cell receptors and induce NK cell apoptosis [37]. On the other hand, NG2, which is present in myofibroblasts and pericytes [38], may improve wound vascularization, serving as a template and guide for infiltrating pro-angiogenic and anti-inflammatory cells, including pericytes, endothelial cells, M2-macrophages, and fibroblasts.

Following wound closure, scar formation and remodeling occurs, with collagens, matrix metalloproteinases, integrins, and soluble mediators playing a central role in this process [6]. Importantly, ADMs were shown to not only support cell adhesion, wound vascularization, and epithelization, but also to reduce scarring [9]. Collagens are the primary component of wound scars, with their deposition and extraction being tightly regulated by myofibroblasts and polarized macrophages, which are, in turn, influenced by the microenvironment. Interestingly, we observed the differentially regulated expression of Col1a1, Col5a3, and Col5a2 in ADM1-treated wounds relative to the remaining ADMs, suggesting less intensive scar formation when compared to the analyzed counterparts. Notably, type V collagens were shown to be produced in the initial stage of fibrosis [39]. Importantly, they co-polymerize with type I collagen fibers and act as a regulator of fibril shape and size [40]. Therefore, the observed downregulation of the abovementioned collagens’ expression in ADM1-treated wounds may, similarly to the proliferative phase, represent a consequence of accelerated kinetic of healing. This hypothesis is also supported by the observed differences in Itga1 (integrin alpha 1 subunit also known as CD49a) and Itga4 (integrin alpha 4, also known as CD49d) expression. Itga1 is a collagen-binding integrin widely expressed on vascular cells, fibroblasts, stromal cells, as well as immune cells [41]. CD49a was suggested to preferably bind type I monomeric collagen fibers compared to the fibrillar forms present in mature connective tissue or scars [42]. Itga4 is a receptor for fibronectin broadly expressed in vascular and immune cells, except for neutrophils [43]. Therefore, the slightly reduced expression of the abovementioned integrin subunits in ADM1-treated wounds observed in this study may be the consequence of a reduced number of immune cells—namely, monocytes/macrophages—which are characteristic contributors in later phases of the scar maturation process [6]. This is also supported by an observed decrease in the expression of Ccl7 (MCP-3) and Ccl12 (MCP-5) chemotactic factors for monocytes/macrophages.

## 5. Conclusions

Taken together, we developed a protocol (ADM1) for abdominoplasty skin decellularization suitable for the preparation of biological dressings. Furthermore, we showed that different decellularization methods affect the purity, structure, and therapeutic properties of ADMs. Importantly, our novel abdominoplasty skin-derived dressing can improve the kinetics of wound closure and scar maturation in the proliferative and remodeling phases of wound healing. Although ADM2 and ADM3 did not improve wound healing kinetics, no adverse effects were observed, proving the safety of abdominoplasty-derived acellular grafts. Given these promising results, a clinical trial is warranted to confirm the beneficial therapeutic effect of our novel dressing in humans. Moreover, further studies are needed to better understand improved wound healing mechanisms induced after the application of our novel abdominoplasty skin-derived dressing.

## Figures and Tables

**Table 1:** Decellularization procedures of abdominoplasty skin.

Decellularization Protocol	Phase 1					Phase 2				Washing			Storage
	Reagents	Time (h)	Temp. (°C)	RPM	Epidermis Removal	Reagents	Time (h)	Temp. (°C)	RPM	Washing/Quantity	Time (h)	(RPM)	Lyophilization/Temp (°C)
ADM1	1M NaCl + antibiotic mix	24	37	40	Mechanical	SDS 0.1% + Antibiotic Mix	24	37	40	H_2_O/5	24	60	YES/−70
ADM2	2M NaCl	24	37	40	Mechanical	SDS 0.1% + Antibiotic Mix	24	37	40	H_2_O/5	24	60	YES/−70
ADM3	TrypLE Select + antibiotic mix	24	37	40	Not Applicable	3% Triton X-100 in PBS + Antibiotic Mix	24	37	40	H_2_O/5	24	60	YES/−70

NaCl—sodium chloride; SDS—sodium dodecyl sulfate; PBS—phosphate-buffered saline, H_2_O—distilled deionized water, 5—five days washing step with daily H_2_O change.

## Data Availability

The data presented in this study are available on reasonable request from the corresponding author. The data shared are in accordance with consent provided by participants on the use of confidential data.

## Supplementary Materials

The following are available online at https://www.mdpi.com/article/10.3390/pharmaceutics13122118/s1, Table S1: Characteristics of antibodies used for immunohistochemical staining’s, Figure S1: Acellular Dermal Matrix 1 (ADM1) and 2 (ADM2) lack immunogenicity, Figure S2: Quantification of the effects of different methods of decellularization on the structure of extracellular matrix components.
