# Supplementary material for: Abdominoplasty Skin-Based Dressing for Deep Wound Treatment—Evaluation of Different Methods of Preparation on Therapeutic Potential
Source: Pharmaceutics. 2021 Dec 8;13(12):2118. doi: 10.3390/pharmaceutics13122118 (PMC8708629; doi:10.3390/pharmaceutics13122118)
Supplement: Supplementary file 1 [file pharmaceutics-13-02118-s001.zip › pharmaceutics-1439108-supplementary.pdf]

pharmaceutics

MDPI

Supplementary Materials: Abdominoplasty Skin-Based Dressing for Deep Wound Treatment—Evaluation of Different Methods of Preparation on Therapeutic Potential

Dawid Groth, Izabela Poplawska, Marlena Tynecka, Kamil Grubczak, Jordan Holl, Aleksandra Starosz, Adrian Janucik, Klaudia Borkowska, Dorota Juchniewicz, Hady Razak Hady, Slawomir Czaban, Joanna Reszec, Artur Kaminski, Tomasz Czech, Cezary Kowalewski, Piotr Fiedor, Zbigniew Zimek, Hanna Lewandowska, Tomasz Oldak, Marcin Moniuszko and Andrzej Eljaszewicz

Table S1. Characteristics of antibodies used for immunohistochemical staining's.

Primary Antibodies			
**Marker**	**Origin/Isotype**	**Clone**	**Supplier**
HLA Class I ABC	Mouse / IgG1	EMR8-5	Abcam
Talin 1 and 2	Mouse / IgG1	8D4	Abcam
NG2	Rabbit / IgG	Polyclonal	Abcam
Vitronectin	Rabbit / IgG	Polyclonal	Abcam
Collagen VII (7a1)	Mouse / IgG1	LH7.2	Thermofisher
Collagen I (1A1)	Rabbit / IgG	Polyclonal	Invitrogen
Collagen III	Rabbit / IgG	Polyclonal	Invitrogen
Collagen IV	Mouse / IgG1	COL-94	Invitrogen

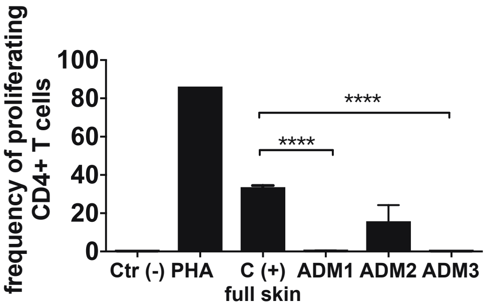

Figure S1. Acellular Dermal Matrix 1 (ADM1) and 2 (ADM2) lack immunogenicity. Summary of analyzes of CD4 T helper cell proliferation after a 7-day incubation with vehicle (Ctr-), phytohemagglutinin (PHA, positive control of proliferation), full-thickness skin (non-irradiated, positive control), ADM1, ADM2, and ADM3. The student's t-test was used. *** $p < 0.001$.

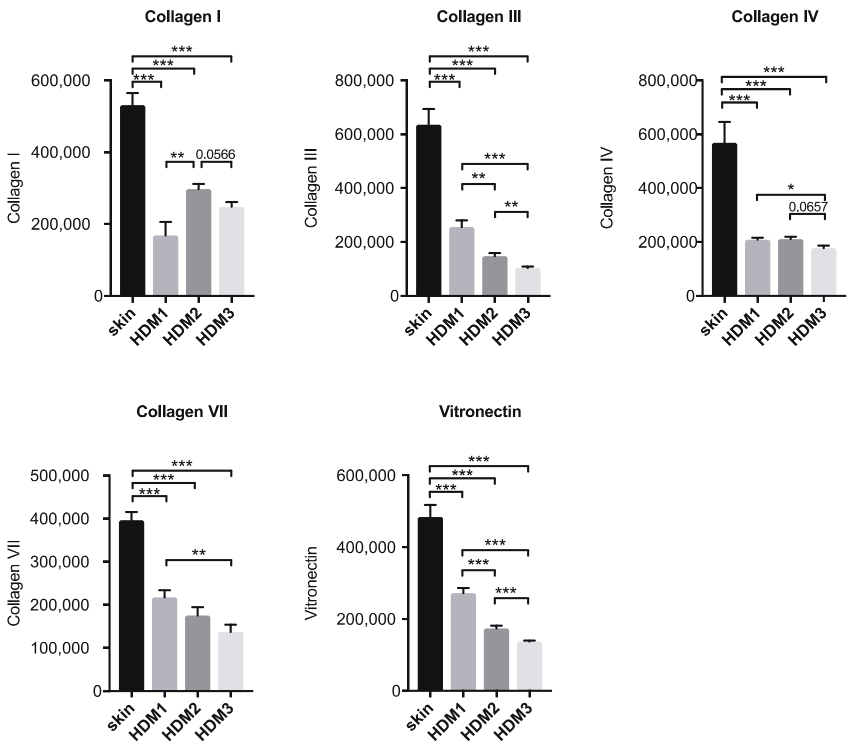

Figure S2. Quantification of the effects of different methods of decellularization on the structure of ex-tracellular matrix components. Summary of quantification analyses of immunohistochemical staining for collagen I, III, IV, VII and vitronectin in in abdominoplasty skin and ADMs derived from abdominoplasty skin decellularized with ADM1, ADM2, or ADM3 protocol. The student's t-test was used. * $p < 0.05$; ** $p < 0.01$; *** $p < 0.001$ (n = 3).
